# The Swedish COVID-19 approach: a scientific dialogue on mitigation policies

**DOI:** 10.3389/fpubh.2023.1206732

**Published:** 2023-07-20

**Authors:** Anders Björkman, Magnus Gisslén, Martin Gullberg, Johnny Ludvigsson

**Affiliations:** ^1^Department of Global Public Health, Karolinska Institutet, Stockholm, Sweden; ^2^Department of Infectious Diseases, University of Gothenburg, Sahlgrenska Academy, Gothenburg, Sweden; ^3^Department of Infectious Diseases, Sahlgrenska University Hospital, Gothenburg, Sweden; ^4^Department of Molecular Biology, University of Umeå, Umeå, Sweden; ^5^Division of Pediatrics, Department of Biomedical and Clinical Sciences, Crown Princess Victoria Children's Hospital, Linköping University, Linköping, Sweden

**Keywords:** COVID-19, SARS-CoV-2, lockdown, excess mortality, scientific dialogue, health policy, Sweden

## Abstract

During the COVID-19 pandemic, Sweden was among the few countries that did not enforce strict lockdown measures but instead relied more on voluntary and sustainable mitigation recommendations. While supported by the majority of Swedes, this approach faced rapid and continuous criticism. Unfortunately, the respectful debate centered around scientific evidence often gave way to mudslinging. However, the available data on excess all-cause mortality rates indicate that Sweden experienced fewer deaths per population unit during the pandemic (2020–2022) than most high-income countries and was comparable to neighboring Nordic countries through the pandemic. An open, objective scientific dialogue is essential for learning and preparing for future outbreaks.

## Key points

- The voluntary, comparatively open policy of the Swedish approach to the COVID-19 pandemic appears to have caused less serious consequences than the lockdown policy used in most countries. However, there may also be other unknown explanations for our findings.
- Learning from the COVID-19 experience is important. Although future pandemics may manifest differently, maintaining an open scientific approach and fostering dialogue will be essential.

## Introduction

During the COVID-19 pandemic, Sweden was among the few countries that did not enforce strict lockdown measures. Instead, the country relied on its citizens' voluntary behavioral changes, considering them to be more sustainable. This approach involved enforcing physical distancing, encouraging working from home, limiting social gatherings and travel, prohibiting most public events, and so on. Initially, masks were mandatory only in healthcare and older adult care settings, but later they were also recommended for crowded public transport. Kindergartens, primary schools, and secondary schools remained open throughout the pandemic, which was a unique policy. A large majority (>90%) of the Swedish population approved, endorsed, and complied with the Swedish policies, according to repeated public polls conducted during the pandemic by the Swedish Civil Contingencies Agency (1).

However, the Swedish approach was heavily criticized by a significant number of scientists at the national (2, 3) and international levels (4, 5) for being too permissive and complacent and, in particular, for keeping schools open and for not legally enforcing mask-wearing in public spaces. That being said, in 2022, opponents of the Swedish policy presented a review of selected publications, largely non-peer-reviewed newspapers, magazines, and reports, which painted a quite negative but scientifically questionable picture of the mitigation and outcome of the Swedish epidemic (6). In contrast, already early in the pandemic, other scientists proposed that the vulnerable groups should be strongly protected but otherwise avoid strict lockdowns (7, 8). The Swedish model has also received support from scientists and was recently considered quite reasonable (9). Unfortunately, the respectful debate regarding the pros and cons of various mitigation policies was often overshadowed by mudslinging and hatred, which even involved scientists (2, 4, 10).

Existing official statistics at both the European and global levels regarding total COVID-19-associated and excess overall mortality rates suggest that Sweden was less affected than most comparable countries that implemented stricter lockdown measures (11–13). Therefore, we summarize the mostly used and referred data on excess all-cause mortality in Sweden and other European countries over the past 3 years (2020–2022).

## Methods

Secondary data were assembled from the websites of Worldometer (11), Our World in Data (12), the Swedish Public Health Agency (14), and the Swedish National Board of Welfare (15). We specifically opted for excess mortality as our measure of choice, considering that the reported COVID-19-associated deaths can vary depending on different definitions of COVID-19 deaths and may include many deaths where COVID-19 was not the cause of death, especially in 2021–2022. Moreover, by examining all-cause mortality, we included deaths that could potentially be indirectly attributed to the negative effects of strict lockdown measures and the overall strain on healthcare systems, leading to reduced access to healthcare for other diseases, among other factors.

## Results

Excess all-cause mortality and estimated degrees of lockdown (intervention index) are presented for 14 selected European countries in Table 1 (12). Among 42 European countries, the cumulative excess all-cause mortality from January 2020 to December 2022 ranged from 46 (Luxembourg) to 1,080 (Bulgaria) deaths per 100,000 inhabitants, with a median of 351/100,000. In Sweden, the excess mortality rate of 158/100,000 was among the lowest, ranked 37th among 42 countries, and not very different from other Nordic countries: Norway (129), Denmark (97), and Finland (228). In most countries, the excess mortality was highest in 2020, before the COVID-19 vaccination was introduced. It was estimated to be 85/100,000 in Sweden, whereas, in Europe, the excess mortality ranged from −9 (Iceland) to 287 (North Macedonia), with a median of 111. The excess mortality in Sweden was thus higher than that in the three neighboring Nordic countries (2, 3, and 26/100,000), partly explained by a higher initial COVID-19 transmission (replication rate), comparable to other European countries (9) and possibly by mortality displacement due to low all-cause mortality in 2019 (16), and perhaps also by poorly organized older adult care structures and an initial lack of protective equipment in these settings (9, 17).

**Table 1:** Excess all-cause mortality (deaths per 100,000 inhabitants) in 14 European countries in relation to the degree of lockdown as estimated by the highest Stringency index (SI) during spring 2020 (Our World in Data).

	**Excess all-cause mortality (deaths per 100,000 inhabitants)**			SI%^*^
	**2020**	**2021–2022**	**2020–2022**	**2020**
Sweden	85	69	158	65
Norway	3	127	129	80
Denmark	2	94	97	72
Finland	26	204	228	85
Belgium	161	100	262	81
France	84	122	207	91
Germany	52	183	241	75
Italy	194	254	451	92
Netherlands	93	164	262	80
Poland	169	294	475	81
Portugal	120	221	273	82
Spain	162	169	332	85
Switzerland	110	106	221	77
United Kingdom	127	153	289	80

^*^Stringency index (%) estimated from a composite measure of 9 community response/restriction indicators such as school closures, workplace closures, travel bans, etc., rescaled to a value from 0 to 100% according to Our World in Data (12).

Interestingly, excess mortality during the second and third years of the pandemic (2021–2022) showed a different profile, with a comparatively low figure for Sweden (69/100,000) compared to the Nordic countries (97–204) and Europe in general (median 192) (12). Only Liechtenstein and Luxembourg had lower excess mortality. Reported COVID-19-associated deaths provide a similar overall picture for Europe although with some significant differences at the individual country level between the reported COVID-19 mortality and the estimated excess mortality (11, 12).

Like many other countries, Sweden largely failed to protect vulnerable older adults, especially before vaccines were rolled out (17). Hence, ~40% of the COVID-19-associated deaths were among patients in nursing homes, and 67% of all COVID-19 deaths were among individuals above 80 years of age, representing 10% of all deaths in that age group. COVID-19 deaths below 50 years of age represented only 1.2% of all COVID deaths, including 21 individuals below 20 years of age, mostly with underlying co-morbidities, representing 1% of all deaths in that age group (15).

## Discussion

Evaluating the outcome of the pandemic and the different mitigation policies is complex and difficult. Excess all-cause mortality during the pandemic, relative to expected mortality, is widely considered a reasonably objective and comparable indicator of both direct and indirect COVID-19-associated deaths (13). It is even more difficult to estimate the overall public health impact in relation to COVID-19 morbidity (“long/post-COVID-19”), mental health effects from lockdown measures, etc. Hence, any data should be interpreted with caution.

The impact of restrictions is not always evident. Intervention policies are thus difficult. Similar to governments in other countries, the Swedish government was aware that strict lockdown measures, such as closing businesses and schools, would have significant social and economic consequences although the main goal was to counteract the pandemic and save lives. In addition, according to Swedish law, politicians cannot govern over independent national authorities such as the Public Health Authority. Current evidence suggests that keeping schools open had limited consequences for the spread of the epidemic and the occurrence of COVID-19 disease, at least in Sweden (18, 19). Furthermore, experts from UNICEF (20) and UNESCO (21) in the present times concurred that school closures had more harmful effects than benefits. A Cochrane meta-analysis revealed that the use of face masks in public space “makes little or no difference” (22), in contrast to many scientists' views, such as those of the Royal Swedish Academy of Sciences (23). Harsh lockdown restrictions may also negatively affect economic and human development, health promotion, and disease prevention, which must be considered when public health consequences are summarized (24). Moreover, several country-specific factors, such as healthcare systems, may influence both COVID-19 transmission (25) and mortality. Hence, what is important and works well in one society may not be optimal in another. Thus, comparisons across countries are inherently difficult and require humility, openness, and objective scientific analysis as evidence becomes available.

In Sweden, excess mortality was especially low from 2021 to 2022, which could be partly due to the high initial mortality rate in 2020 among frail older adults in nursing homes, with a short remaining life expectancy. The fact that numerous countries also experienced significant excess mortality in 2021–2022 may suggest that strict lockdown may have caused negative indirect health effects. It is also possible that the voluntary measures implemented in Sweden were more sustainable and/or that the establishment of protective immunity from previous COVID-19 infection and/or vaccination was not as widespread.

This study also has some limitations. Countries may differ in how they report and quantify the factors that contribute to the lockdown stringency index. Additionally, mortality rates in the years before the pandemic may influence the estimation of excess death rates during the epidemic. Notably, our comparisons are limited to European countries, and the findings may be less relevant for non-European countries with very different structures and populations.

Even though our presented results suggest that strict lockdowns of society may not be the most effective approach and could potentially have counterproductive effects, it is important to exercise caution when drawing practical implications from our analyses. Conclusions regarding future approaches to epidemics should be based on more comprehensive studies that are tailored to different regions and various types of infectious agents.

In conclusion, Sweden experienced relatively fewer deaths per population unit than most other high-income countries that implemented stricter lockdown measures. It is concerning that some scientists who advocated for stringent measures seem to disregard real-world data and cling to their version of reality. The ability to learn from mistakes and acknowledge that hypotheses may be wrong is essential for future pandemic preparedness. This, coupled with careful analysis, is crucial for developing effective strategies in the face of future outbreaks.

## Data availability statement

The original contributions presented in the study are included in the article/supplementary material, further inquiries can be directed to the corresponding author.

## Author contributions

JL conceived the idea, contributed to data collection, and wrote the first draft of the manuscript. AB contributed to data collection. All authors critically reviewed the manuscript and approved its final version.
